# Kasabach-Merritt Phenomenon of the Parotid Gland: Case Report and Literature Review

**DOI:** 10.7759/cureus.80909

**Published:** 2025-03-20

**Authors:** Jose D Cardona Ortegón, Laura M Olarte Bermudez, Laura Morales, Andres Francisco Vasquez Perdomo, Maria Jose Cardona Ortegón, Hernan D Paez

**Affiliations:** 1 Radiology, University Hospital Fundación Santa Fé de Bogotá, Bogotá, COL; 2 Radiology, Universidad El Bosque, Bogotá, COL; 3 Radiology, Universidad de la Sabana, Bogotá, COL; 4 Radiology, University Hospital Fundación Santa Fe de Bogotá, Bogotá, COL; 5 Radiology, University Hospital Fundación Santa Fé de Bogotá, Bogota, COL; 6 School of Medicine, Universidad de la Sabana, Bogotá, COL

**Keywords:** angioma, diagnostic imaging, kasabach-merritt phenomenon, newborn, parotid gland, vascular tumor

## Abstract

Kasabach-Merritt phenomenon (KMP) is a rare but life-threatening condition characterized by consumptive coagulopathy associated with a vascular tumor. This phenomenon usually presents in early infancy and commonly reported sites of tumor include extremities, trunk, and neck. We report a newborn with KMP, presenting with parotid gland hemangioma and associated vascular complications. This case report and literature review, aim to describe the imaging features of KMP in an infant, along with a compilation of cases from the literature, with a particular focus on the radiological findings of the vascular tumor. Sixteen patients described in seven articles published between 1993 and 2021 were analyzed. Most of the cases were males (56%), with a median age of 13.5 days, an interquartile range (IQR) of 2.75 to 30 days, and lesions primarily located in the parotid gland. The most commonly affected side was 87.5% of cases involved the main gland, 6.25% the thoracic wall and 6.25 the supraclavicular region. The lesions also involved secondary sites such as the temporal region, submandibular area, neck, facial region, and extensive areas involving the thorax and extremities. KMP in the parotid gland is a rare manifestation. Advanced radiological imaging, particularly MRI and contrast-enhanced CT, plays a critical role in the diagnosis, staging, and detection of associated complications. The integration of imaging findings with clinical and laboratory data is essential for timely therapeutic decisions. Continued efforts to refine imaging protocols and gather epidemiological data will improve the prognosis of patients with this complex condition, emphasizing the need for a multidisciplinary approach.

## Introduction

The Kasabach-Merritt phenomenon (KMP) is a rare but life-threatening condition characterized by consumptive coagulopathy associated with a vascular tumor. KMP usually presents in early infancy with an incidence of 0.07 per 100,000 children annually ​[1]​. It is characterized by intravascular coagulopathy, likely due to platelet sequestration within the lesion and activation of coagulation cascades, and has a high mortality rate due to hemorrhage, massive blood loss, shock, intracranial bleeding, or other internal hemorrhages, with mortality rates reaching up to 30% ​​[2]. Clinically, it presents as an enlarging vascular lesion of infancy, associated with thrombocytopenia, microangiopathic hemolytic anemia, and consumptive coagulopathy ​[1]​. The International Society for the Study of Vascular Anomalies (ISSVA) divides vascular malformations into two categories: vascular tumors with proliferative changes of endothelial cells, and vascular anomalies [3]. Recent studies have shown that KMP is almost exclusively associated with two uncommon vascular tumors, kaposiform hemangioendotheliomas (KHE) and tufted angiomas [1]. Both are rare tumors, and their exact incidence is unknown. They need to be differentiated from infantile hemangiomas.

The diagnostic challenge requires an early diagnosis, based on recognition of clinical features and laboratory tests that reveal severe thrombocytopenia, hypofibrinogenemia, anemia, and elevated D-dimer levels ​[1]. There are different imaging modalities like ultrasound (US) and magnetic resonance imaging (MRI) that can assess and establish the vascular nature of the lesions, exclude other differential diagnoses and evaluate the relation with adjacent anatomical structures. In this case, we report a newborn with KMP, presenting in the early neonatal period with parotid gland hemangioma and associated vascular complications. Following this, we conducted a literature review that encompassed cases presenting with KMP with parotid gland involvement.

## Case presentation

**Figure 1:**
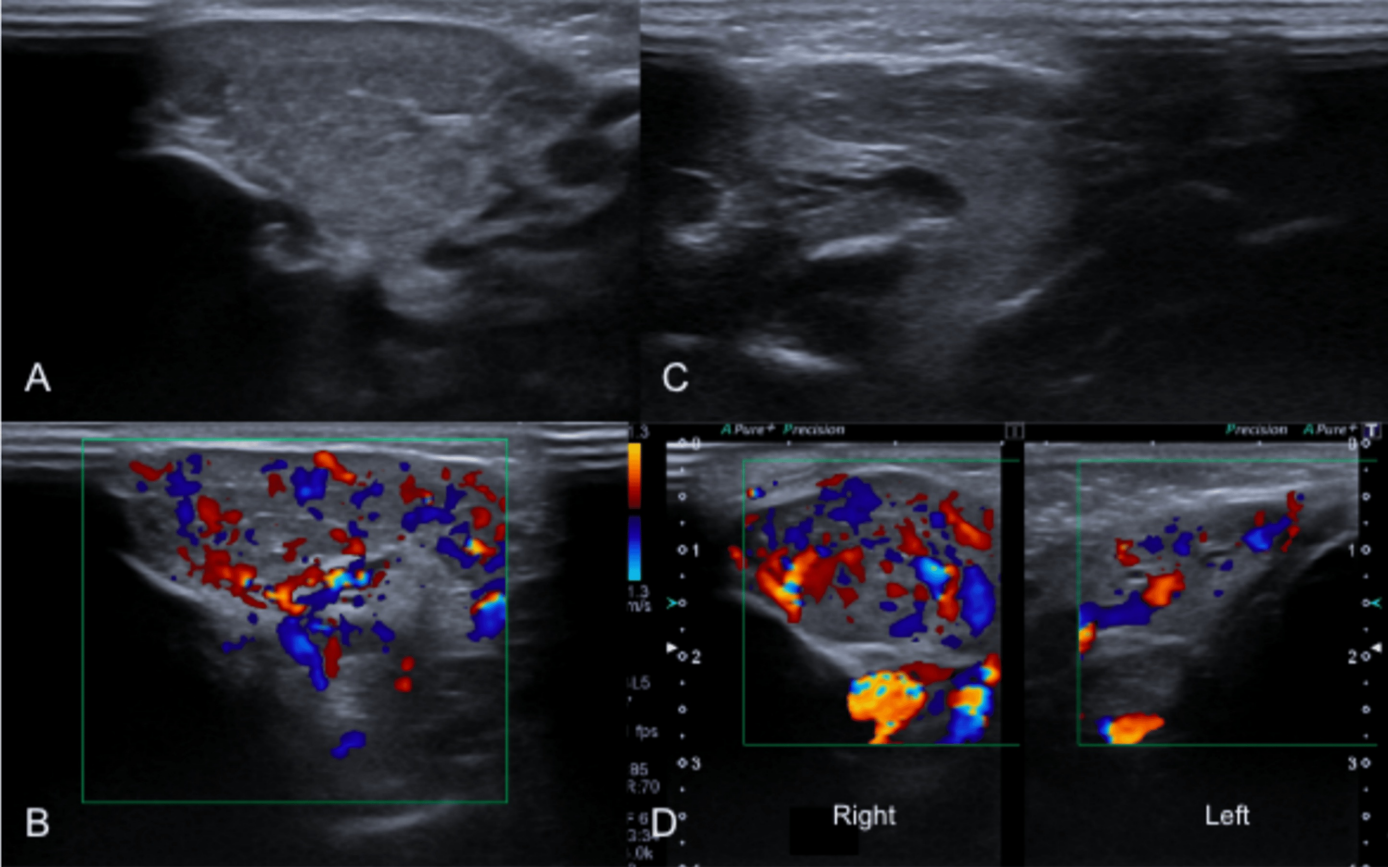
Soft tissue ultrasound of face. (A) The right parotid gland is diffusely enlarged and hypoechoic. (B) The color Doppler shows prominent internal vascularity. (C) Left parotid gland was normal. (D) In color Doppler there is a comparative increase of vascular flow in the right parotid gland compared to the left.

**Figure 2:**
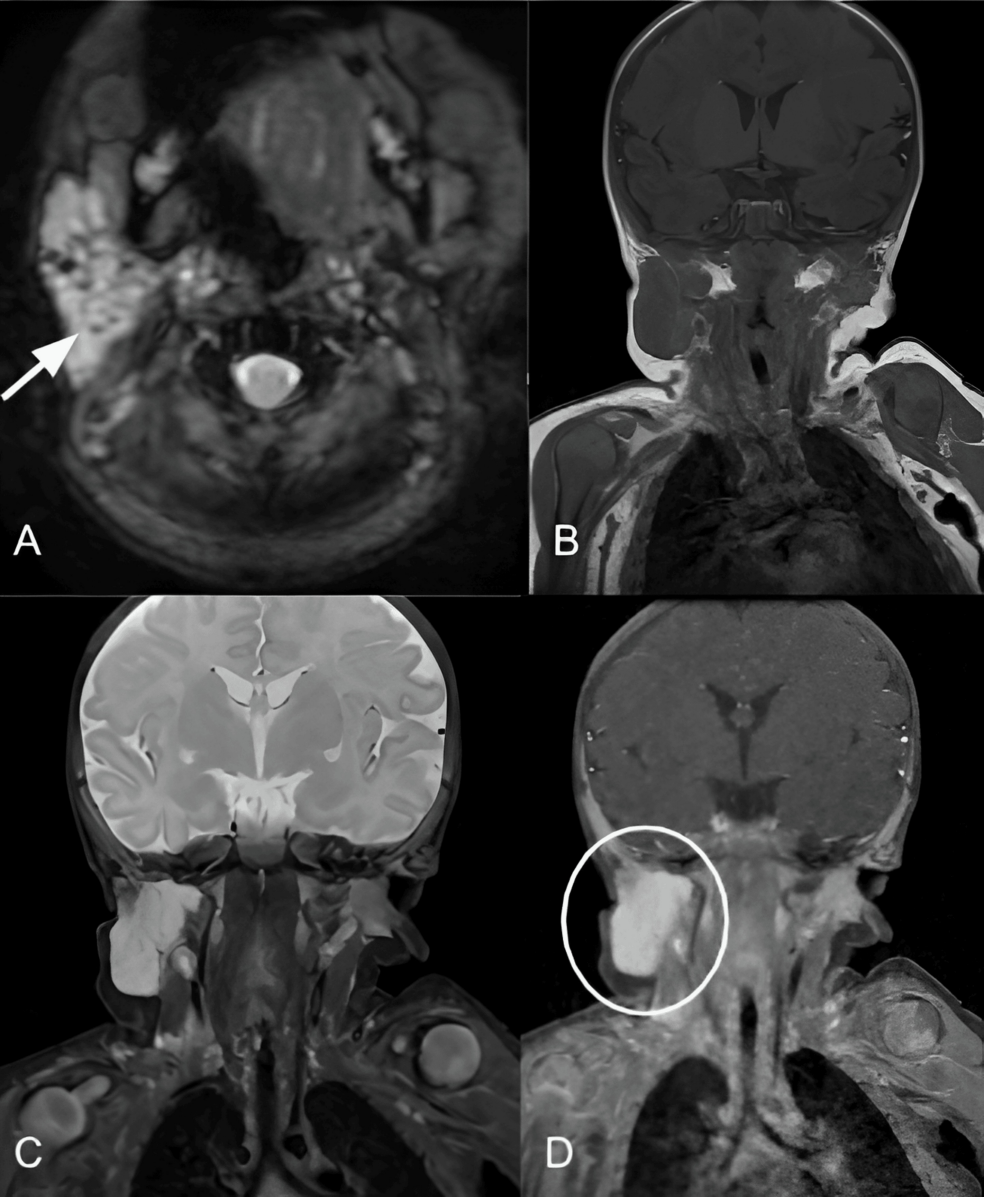
MRI of the Brain and Face. Axial T2-weighted (A), coronal T1- (B), T2-weighted (C) and gadolinium-enhanced image (D) showed a lobulated mass centered in the right parotid gland, hypointense in T1, hyperintense in T2 with homogeneously strong and early enhancement. Notice the prominent flow voids within the gland which correspond to vessels (arrow in A). No infiltration of the surrounding structures.

A one-month-old female presented to the emergency department with a three-day history of painful swelling over the right half of her face and petechiae on the facial and right mandibular angle. This was an acute first episode, triggered by minor trauma. Initial laboratory tests revealed thrombocytopenia (platelet count of 6,000 per mm3) and anemia (hemoglobin: 7.3 mg/dL); thus, requiring platelet transfusion. Targeted ultrasound shows an enlarged right parotid gland with marked internal vascularity (Figure 1). Neck MRI evidenced a soft tissue mass within the right parotid gland, involving both the superficial and deep lobes, hyperintense in T2 with flow voids and homogeneous enhancement on postcontrast images (Figure 2). The most probable diagnosis was vascular malformation, most likely infantile hemangioma. 

**Figure 3:**
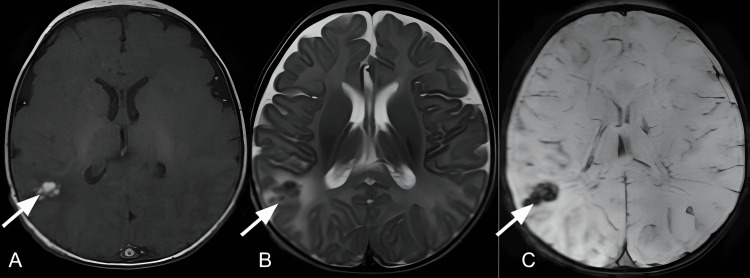
Brain MRI Axial T1- (A), T2-weighted images (B) and susceptibility weighted imaging (C) showed a right parietal subcortical hematoma with mild surrounding vasogenic edema.

**Figure 4:**
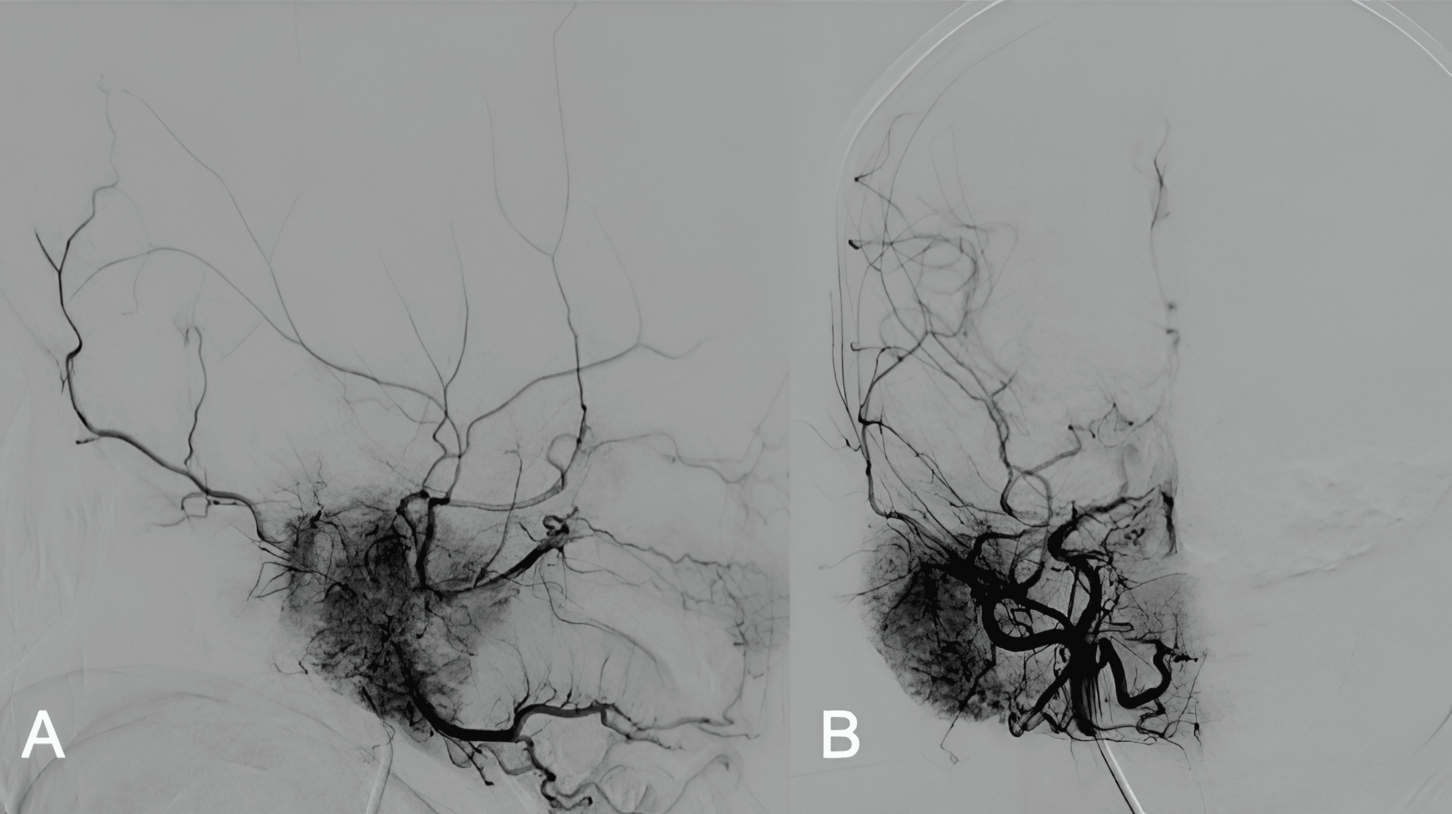
Brain Angiography lateral (A) and anteroposterior (B) views showed a large mass of pyramidal morphology in the right parotid region, with increased vascular blush in the right parotid lesion without arteriovenous shunt, measuring 58 x 51 x 35 mm, in relation to hemangioma.

One month later, the patient presented with persistent facial swelling, irritability and vomiting, without respiratory symptoms or difficulty breathing suggesting increased intracranial pressure. On physical examination, new bilateral facial petechiae were noted but no focal neurological deficits were noted. Laboratories showed significant platelet consumption leading to severe thrombocytopenia. To exclude other potential causes of thrombocytopenia, additional laboratory tests were requested including infectious disease screening and immunologic tests, all of which were negative. Due to the high risk of bleeding in the central nervous system, brain MRI was performed showing a right parietal intracranial hematoma and multiple intraparenchymal hemorrhagic foci in both cerebellar hemispheres (Figure 3). Subsequently, cerebral angiography was performed to rule out the presence of a brain vascular malformation, which showed no evidence of malformations and confirmed the presence of the right parotid hemangioma (Figure 4).

## Discussion

A compilation of cases from the literature of KMP with parotid gland involvement is shown in Table 1.

**Table 1:** Compilation of cases from the literature of Kasabach Merritt phenomenon with parotid gland involvement according to year of publication, sex, age, anatomic region affected (in addition to the parotid gland), diagnostic imaging modality and imaging findings (if documented). Sources: ​[4-10]

Authors	Year	No. of patients	Sex	Age	Anatomic location	Imaging modality	Imaging findings
Mukerji et al.	2009	1	Female	1 month	Right parotid gland	MRI	Poorly defined enhancing lesion involving the right face, neck, right jugular vein and impinging on the airway.
Su L, Wang D, Fan X	2015	6	3 males and 3 females	3-40 days	Temporal region (16,7%), parotid region (33,3%), submandibular region and neck (50%)	CT and MRI to ascertain the involvement of adjacent structures	N/A
Duclaux-Loras et al.	2015	2	Males	a. 2 months b.15 days old	a. Left cervico-occipito-scapular region with parotid extension. b. Left shoulder with extension on the inferior hemithorax and left arm.	MRI in one case (a).	Angiomatous lesion of the cervico-occipito-scapular region beyond the median line with invasion of the pharyngeal region without compression of the upper airway
Takato T, Komuro Y, Yonehara Y	1993	1	Male	1 month	Left parotid region extending into the upper cervical region	CT	Large swelling of the left parotid gland
Arunachalam P, Ravi-Kumar VR, Swathi D	2012	4	2 male 2 female	Newborn to 4 days	Right parotid, chest wall, right arm, left thigh	US: 3 cases MRI: 2 cases	US: heterogeneous mass with increased vascularity. MRI: diffuse, large circumferential, soft tissue mass lesion.
Yadav D et al.	2021	1	Male	6 day	Right half face, extending from angle of the mandible to scalp	US, CT and MR angiography	N/A
González de Dios et al.	1996	1	Female	Newborn	Left parotid region	N/A	N/A

A total of 16 patients described in seven articles published between 1993 and 2021 were analyzed. Most of the cases were males (56%), with a median age of 21.5 days (range from newborn to two months). 

Sonography in only one of the articles ​[8]​ demonstrated a heterogeneous mass with markedly increased vascularity, as reported in our case. Contrast-enhanced MRI was the most commonly used diagnostic modality, and it was highly relevant to characterize the vascular nature of the lesion, exclude other differential diagnosis and define its local extension. The requirement of biopsy was limited in the studies due to the high risk of hemorrhage.

Kasabach-Merritt phenomenon is a rare condition characterized by a proliferative vascular lesion in infants, consumption coagulopathy, and thrombocytopenia. Usually, it presents within the first year of life [2,8]. Lesions associated with KMP are often kaposiform hemangioendotheliomas and, less frequently, other forms of complex hemangiomas. The most common locations for these hemangiomas include the extremities (53%), followed by the trunk (15%), retroperitoneum (15%), and, to a lesser extent, the neck (7.6%) and spleen (7.6%) [11]. Other studies have reported a higher frequency in the retroperitoneum, cervicofacial region, and upper extremities [12]. Involvement of the parotid gland is exceedingly rare. Like our case, the literature describes 16 patients with parotid gland involvement. The analysis of these cases revealed that most patients were males (56%), with a median age of presentation of 21.5 days, and an age range extending from the neonatal period to two months of life. In a study of 16 patients, the distribution of Kasabach-Merritt cases indicated that 37.5% presented on the right side, while 50% were localized on the left side. Additionally, 6.25% of the cases were identified in the thoracic wall and another 6.25% in the supraclavicular region. These findings suggest a greater frequency of involvement on the left side.

Pathophysiology of the Kasabach-Merritt phenomenon

The KMP involves endothelial defects within the vascular architecture of the lesion, which trigger a cascade of platelet activation and coagulopathy [9,13,14]. These vascular lesions are characterized by an abnormal endothelium that activates platelets and facilitates the formation of intralesional thrombi comprised of platelets and fibrin. This persistent thrombosis leads to the consumption of coagulation factors, platelets, and fibrinogen, resulting in a consumptive coagulopathy [15-17]. At the molecular level, there is a significant increase in pro-inflammatory cytokines and angiogenic growth factors, including vascular endothelial growth factor (VEGF) and interleukin-6 (IL-6), which contribute to endothelial dysfunction and the progression of coagulopathy [4]. Additionally, enhanced fibrinolysis, due to plasmin activation as a response to sustained thrombosis, exacerbates the bleeding diathesis and perpetuates the state of consumptive hemostatic factors [9,14].

Clinical assessment

Physical Examination

The physical examination is crucial for the timely suspicion and diagnosis of KMP. Clinically, this condition should be suspected in the presence of a growing, painful, and firm mass accompanied by superficial or visceral hemorrhagic manifestations. Patients typically exhibit cutaneous or subcutaneous lesions characterized by purplish or reddish pigmentation, edema, and tenderness, which may involve the skin, subcutaneous tissue, and muscles [12]. These lesions often demonstrate rapid growth and are frequently associated with local or systemic bleeding signs [6]. Although KMP lesions are predominantly superficial, they may, in certain instances, extend to infiltrate internal structures [13]. In such cases, involvement of deeper organs may manifest as abdominal pain and distension, intestinal obstruction, or, in instances of mediastinal or pharyngeal involvement, respiratory distress [13].

Laboratory Studies

Laboratory assessments are essential for identifying the consumptive coagulopathy associated with this phenomenon, characterized by findings such as elevated D-dimer levels, which indicate active fibrinolysis and the continuous consumption of coagulation factors due to thrombus formation and degradation within the lesion [9]. Severe thrombocytopenia is a common finding, resulting from the sequestration and destruction of platelets in the vascular lesion, significantly increasing the risk of hemorrhage [14]. Hemolytic anemia is also prevalent, attributed to the destruction of erythrocytes. Hypofibrinogenemia, resulting from excessive consumption of fibrinogen during the coagulation cascade, predisposes patients to severe local and systemic bleeding [14]. Additionally, prolonged prothrombin time (PT) and activated partial thromboplastin time (aPTT) reflect the consumption of multiple coagulation factors, including factors V, VIII, and XII, indicative of disseminated intravascular coagulation (DIC) secondary to KMP [1]. This process leads to a generalized reduction in essential coagulation factors, worsening the bleeding tendency and challenging the clinical management of the patient.

Diagnostic imaging modalities

Ultrasound (US) 

US is the first-line modality for the initial evaluation of superficial lesions. Its accessibility, cost-effectiveness, and absence of ionizing radiation make it particularly suitable for pediatric use. US findings of vascular lesions display a range of characteristics but typically present as hypoechoic or heterogeneously echogenic lesions with irregular margins and a mixed echotexture, which may indicate necrosis, hemorrhage, or intralesional fibrosis [16,18]. US also facilitates the assessment of the relation between the lesion with surrounding structures and its invasion into muscle layers. Color and spectral Doppler US are particularly essential for evaluating the vascularization of the lesion. These techniques can demonstrate patterns of low-resistance and high-velocity flow, characteristic of vascular tumors. The identification of nutrient vessels may suggest an aggressive behavior of the hemangioma, in addition to aiding in the guidance of surgical or endovascular treatment approaches [16].

Computed Tomography (CT)

This modality is particularly useful for evaluating the extent and internal characteristics of deeper lesions or those involving internal structures, such as the retroperitoneum or thoracic cavity. CT offers precise anatomical details, emphasizing the morphology of lesions and their relationships with adjacent structures [19]. The most common findings include a heterogeneous enhancing mass, reflecting a mixture of highly vascularized areas along with regions of necrosis, thrombosis, or fibrosis. Typically, a complex vascular network within the tumor is observed. Detailed assessment of enhancement, including analysis of its timing and pattern, facilitates the differentiation between hemangiomas and other vascular lesions, such as arteriovenous malformations. Intralesional calcifications are rare in hemangiomas but may be present in chronic lesions that have experienced previous hemorrhages [19,10]. CT is not routinely used for evaluation of suspected vascular tumours or malformations located in extremities of superficial spaces, it is performed only to evaluate focal lesions in solid organs and is best avoided in infants to avoid radiation exposure. CT also allows for the assessment of compression on vital structures, which is critical in lesions affecting areas such as the mediastinum, where compression of the trachea, major vessels, or neural structures can have significant clinical implications [20].

Magnetic Resonance Imaging (MRI)

MRI is the modality of choice for the diagnosis and follow-up of vascular tumors due to its ability to characterize soft tissues, vascularization, and the complex anatomical relationships of these lesions. It provides high-resolution images without employing ionizing radiation, making it especially valuable in the pediatric population, with the only clear drawback being its availability. Contrast-enhanced magnetic resonance angiography (MRA) is often used instead of time-of-flight MRA (TOF-MRA). In T1-weighted sequences, vascular tumours are typically isointense or slightly hypointense compared to muscle, indicating a dense cellular content and the presence of soft tissue. The administration of contrast medium highlights the highly vascular nature of the lesion, demonstrating intense and heterogeneous enhancement that is crucial for confirming its diagnosis and assessing its extent [15,16]. In T2-weighted images, the lesions are usually markedly hyperintense, reflecting the high concentration of fluid content, which is particularly useful for differentiating vascular tumors from other lesions such as fibromas or sarcomas [15]. Fat-suppressed sequences enhance the contrast between the lesion and surrounding tissues by suppressing the signal from fat, being especially useful in areas with abundant fat, such as the neck or mediastinum [18].

Finally, MR angiographic sequences allow detailed visualization of the internal vascular architecture, including nutrient vessels and their relationship with large-caliber vascular structures, revealing tortuous and dilated arteries characteristic of complex hemangiomas [9]. Imaging modalities have a significant role in the assessment of patients with KMP particularly those with masses in the head and neck region. MRI is regarded as the modality of choice due to its ability to provide detailed characterization of soft tissues and vascularization-key components in the diagnosis and monitoring of these complex lesions. However, it is concerning that many cases do not undergo MRI prior to treatment, potentially leading to misdiagnosis and suboptimal management. This situation underscores the necessity to optimize the diagnostic workflow by prioritizing early MRI evaluation when a vascular lesion is suspected in a patient with coagulopathy, thus facilitating more effective and accurate patient care.

Treatment

KMP requires a multidisciplinary approach, tailored to the specific characteristics of each patient. Treatment options range from palliative care and local therapies to pharmacological and surgical management. The initial therapy focuses on addressing the coagulopathy of consumption, which may include platelet transfusions and coagulation factors to stabilize the patient [21,22]. Some studies suggest the use of high-dose steroids, chemotherapy (such as cyclophosphamide), antiplatelet therapy (like aspirin), as well as propranolol, embolization, radiotherapy, and sclerotherapy as adjunctive treatments [23]. Surgical excision is considered a crucial component of the therapeutic strategy, as it aims to remove the vascular lesion responsible for the coagulopathy [24,25]. This intervention not only alleviates symptoms and enhances the patient’s quality of life but also contributes to the resolution of the hemostatic dysfunction associated with the syndrome. Thus, a multidisciplinary approach involving pediatricians, surgeons, and radiologists is essential to ensure optimal outcomes in the diagnosis and management of this complex condition.

## Conclusions

KMP in the parotid gland is a rare manifestation that poses complex diagnostic and therapeutic challenges. MRI and contrast-enhanced CT are essential tools to accurately characterize the extent and vascular nature of the lesion, allowing differentiation from other tumors and optimizing therapeutic decisions. Further studies are required to establish advanced and standardized imaging protocols that allow a comprehensive approach, aimed at early detection and treatment of the lesion, to reduce the risk of coagulopathy in these patients.
